# Association of tumor TROP2 expression with prognosis varies among lung cancer subtypes

**DOI:** 10.18632/oncotarget.15647

**Published:** 2017-02-23

**Authors:** Kentaro Inamura, Yusuke Yokouchi, Maki Kobayashi, Hironori Ninomiya, Rie Sakakibara, Sophia Subat, Hiroko Nagano, Kimie Nomura, Sakae Okumura, Tomoko Shibutani, Yuichi Ishikawa

**Affiliations:** ^1^ Division of Pathology, The Cancer Institute, Department of Pathology, The Cancer Institute Hospital, Japanese Foundation for Cancer Research, Koto-ku, Tokyo 135-8550, Japan; ^2^ Translational Medicine & Clinical Pharmacology Department, Daiichi Sankyo Co., Ltd., Shinagawa-ku, Tokyo 140-0005, Japan; ^3^ Department of Integrated Pulmonology, Tokyo Medical and Dental University, Bunkyo-ku, Tokyo 113-8519, Japan; ^4^ Thoracic Oncology Center, The Cancer Institute Hospital, Japanese Foundation for Cancer Research, Koto-ku, Tokyo 135-8550, Japan

**Keywords:** antibody-drug conjugate, lung cancer, molecular targeted therapy, outcome, TROP2

## Abstract

TROP2 is a transmembrane glycoprotein that is overexpressed in various cancers. Emerging evidence suggests that TROP2-targeting therapies are efficacious and safe in patients with multiple prior treatments. TROP2 is a promising target for lung cancer treatment; however, little is known regarding the association of TROP2 expression with clinicopathological/molecular features, including prognosis, in lung cancer. We examined consecutive cases of adenocarcinoma, squamous cell carcinoma (SqCC), and high-grade neuroendocrine tumor (HGNET) for the membranous expression of TROP2 using immunohistochemistry. High TROP2 expression was observed in 64% (172/270) of adenocarcinomas, 75% (150/201) of SqCCs, and 18% (21/115) of HGNETs. Intriguingly, the association of TROP2 expression with mortality was dependent on the lung cancer subtype. High TROP2 expression was associated with higher lung cancer-specific mortality in adenocarcinomas [univariable hazard ratio (HR) = 1.60, 95% confidence interval (CI) = 1.07–2.44, *P* = 0.022)], but not in SqCCs (univariable HR = 0.79, 95% CI = 0.35–1.94, *P* = 0.79). In HGNETs, high TROP2 expression was associated with lower lung cancer-specific mortality in both univariable and multivariable analyses (multivariable HR = 0.13, 95% CI = 0.020–0.44, *P* = 0.0003). Our results suggest a differential role for TROP2 in different lung cancer subtypes.

## INTRODUCTION

TROP2 (also known as TACSTD2) is a transmembrane glycoprotein that has high expression in many cancers and is associated with patient survival [1–11]. Emerging evidence suggests that TROP2 is a promising molecular target for the treatment of various malignancies [11]. Several ongoing clinical trials for TROP2-targeting therapies are showing signs of efficacy [11]. TROP2 has been used as a target of antibody-drug conjugate (ADC) therapy [11]. Sacituzumab govitecan (IMMU-132) is an anti-TROP2 ADC [11–18] that contains SN-38, the active metabolite of irinotecan. Without severe side effects, IMMU-132 has been effective against triple-negative breast cancer [19], metastatic small cell lung carcinoma (SCLC) [14], and metastatic non-SCLC (NSCLC) resistant to anti-PD-1/PD-L1 therapy [15].

Lung carcinoma represents a group of histologically and molecularly heterogeneous diseases [20–28]. The major subtypes include adenocarcinoma, squamous cell carcinoma (SqCC), and high-grade neuroendocrine tumor (HGNET), which consists of SCLC and large cell neuroendocrine carcinoma (LCNEC). Even within the same subtype, tumors display heterogeneous characteristics. For example, alterations in cancer driver genes differ between lung adenocarcinomas (e.g., *EGFR*, *KRAS*, *ALK*, *RET*, and *ROS1*) [27]. Little is known about the role of TROP2 in lung cancer [1–3, 11, 14, 15]. Because TROP2 is a promising molecular target for the treatment of lung cancer, we examined the association of TROP2 expression with clinicopathological and molecular features as well as with prognosis of various lung cancer subtypes, including 270 consecutive cases of adenocarcinoma, 201 cases of SqCC, and 115 cases of HGNET (74 cases of SCLC and 41 cases of LCNEC).

## RESULTS

### TROP2 expression in lung cancer

**Figure 1:**
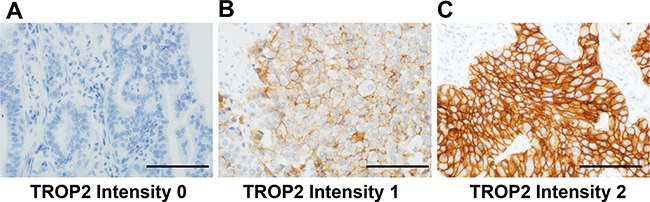
Immunohistochemical evaluation of membranous TROP2 expression in tumor cells from patients with lung adenocarcinoma **(A)** TROP2 intensity 0 (negative), **(B)** TROP2 intensity 1 (weak to moderate), and **(C)** TROP2 intensity 2 (strong). Figure 1A shows a TROP2 immunohistochemical image of well- to moderately-differentiated adenocarcinoma, whereas Figure 1B and 1C show those of poorly-differentiated adenocarcinoma. Scale bar = 200 μm.

We defined high TROP2 expression as intensity 1 with ≥50% expression or intensity 2 with ≥10% expression in tumor membranous staining, as mentioned in Materials and Methods section and presented in Figure 1. High TROP2 expression was observed in 172 (64%) adenocarcinomas, 150 (75%) SqCCs, and 21 (18%) HGNETs using immunohistochemistry. Table 1 shows the clinicopathological and molecular characteristics of each lung cancer subtype according to the TROP2 expression level (no/low vs. high). In adenocarcinoma, high TROP2 expression was associated with the male gender (*P* = 0.0018), larger tumor size (>30 mm) (*P* = 0.016), higher pathological-stage (p-stage) (II–IV) (*P* = 0.012), and less tumor differentiation (moderate to poor) (*P* = 0.045). In SqCC, high TROP2 expression was associated with a higher rate of well tumor differentiation (*P* = 0.040). In HGNET, high TROP2 expression was associated with the LCNEC subtype (*P* = 0.0013). Of the 115 cases of HGNETs, only 22 cases of SCLC underwent neoadjuvant chemotherapy, and high TROP2 expression was not associated with the status of neoadjuvant chemotherapy in SCLC (*P* = 0.67). In HGNET, the Ki-67 index (< 60% vs. ≥ 60%) was not associated with TROP2 expression (no/low vs. high expression) (*P* = 0.61).

**Table 1:** Clinicopathological and molecular characteristics of lung cancer according to TROP2 expression in tumor cells

Variables	Adenocarcinoma				SqCC				HGNET			
	*N* of samples (%)	TROP2 expression			*N* of samples (%)	TROP2 expression			*N* of samples (%)	TROP2 expression		
		No/low (n=98) (36%)	High (n=172) (64%)	*P*-values		No/low (n=51) (25%)	High (n=150) (75%)	*P*-values		No/low (n=94) (82%)	High (n=21) (18%)	*P*-values
Age (years)				0.24				0.39				0.78
< 60	96 (36%)	33 (34%)	63 (37%)		21 (10%)	7 (14%)	14 (9.3%)		28 (24%)	24 (26%)	4 (19%)	
≥ 60	174 (64%)	65 (66%)	109 (60%)		180 (90%)	44 (86%)	136 (91%)		87 (76%)	70 (74%)	17 (81%)	
Gender				0.0018				0.84				0.76
Male	144 (53%)	40 (41%)	104 (60%)		175 (87%)	44 (86%)	131 (87%)		93 (81%)	75 (80%)	18 (86%)	
Female	126 (47%)	58 (59%)	68 (40%)		26 (13%)	7 (14%)	19 (13%)		22 (19%)	19 (20%)	3 (14%)	
Smoking status				0.17				0.45				1.00
Never smoker	112 (41%)	46 (47%)	66 (38%)		2 (1.0%)	1 (2.0%)	1 (0.7%)		3 (2.6%)	3 (3.2%)	0 (0%)	
Ever smoker	158 (59%)	52 (53%)	52 (53%)		198 (99%)	50 (98%)	148 (99%)		112 (97%)	91 (97%)	21 (100%)	
Smoking Index (SI)				0.36				1.00				1.00
SI < 400	150 (56%)	58 (59%)	92 (53%)		13 (6.5%)	3 (5.9%)	10 (6.7%)		18 (16%)	15 (16%)	3 (14%)	
SI ≥ 400	120 (44%)	40 (41%)	80 (47%)		187 (94%)	48 (94%)	139 (93%)		97 (84%)	79 (84%)	18 (86%)	
Tumor size				0.016				0.79				0.20
≤ 30 mm	150 (56%)	64 (65%)	86 (50%)		82 (41%)	20 (39%)	62 (41%)		69 (60%)	59 (63%)	10 (48%)	
> 30 mm	119 (44%)	34 (35%)	85 (50%)		119 (59%)	31 (61%)	88 (59%)		46 (40%)	35 (37%)	11 (52%)	
p-stage				0.012				0.67				0.39
I	152 (56%)	65 (66%)	87 (51%)		117 (58%)	31 (61%)	86 (57%)		53 (47%)	45 (48%)	8 (38%)	
II–IV	118 (44%)	33 (34%)	85 (49%)		84 (42%)	20 (39%)	64 (43%)		61 (54%)	48 (52%)	13 (62%)	
Tumor differentiation				0.045				0.040				
Well	113 (42%)	49 (50%)	64 (37%)		17 (8.6%)	1 (2.0%)	16 (11%)					
Moderate to poor	156 (58%)	49 (50%)	107 (63%)		180 (91%)	49 (98%)	131 (91%)					
SCLC or LCNEC												0.0013
SCLC									74 (64%)	67 (71%)	7 (33%)	
LCNEC									41 (36%)	27 (29%)	14 (67%)	
*EGFR* status				0.26								
Wild type	98 (51%)	32 (46%)	66 (54%)									
Mutant	94 (49%)	38 (54%)	56 (46%)									
*KRAS* status				0.89								
Wild type	168 (88%)	61 (88%)	107 (88%)									
Mutant	23 (12%)	8 (12%)	15 (12%)									
*ALK* rearrangement				0.75								
Negative	260 (96%)	95 (97%)	165 (96%)									
Positive	10 (3.7%)	3 (3.1%)	7 (4.1)									
Neoadjuvant chemotherapy												0.072
No	270 (100%)	98 (100%)	172 (100%)		201 (100%)	51 (100%)	150 (100%)		93 (81%)	73 (78%)	20 (95%)	
Yes	0 (0%)	0 (0%)	0 (0%)		0 (0%)	0 (0%)	0 (0%)		22 (19%)	21 (22%)	1 (4.8%)	
Adjuvant chemotherapy				NA				NA				0.16
No	NA	NA	NA		NA	NA	NA		50 (43%)	38 (40%)	12 (57%)	
Yes	NA	NA	NA		NA	NA	NA		65 (57%)	56 (60%)	9 (43%)	
Ki-67 index												0.61
< 60%									55 (48%)	46 (49%)	9 (43%)	
≥ 60%									60 (52%)	48 (51%)	12 (57%)	

The percentages indicate the proportion of cases with a specific clinical, pathological, or molecular feature within each category.

Abbreviations: HGNET, high-grade neuroendocrine tumor; LCNEC, large cell neuroendocrine carcinoma; p-stage, pathological stage; SCLC, small cell lung carcinoma; SI, smoking index = (number of cigarettes per day) × (duration in years); SqCC, squamous cell carcinoma.

### TROP2 expression and lung cancer mortality

**Figure 2:**
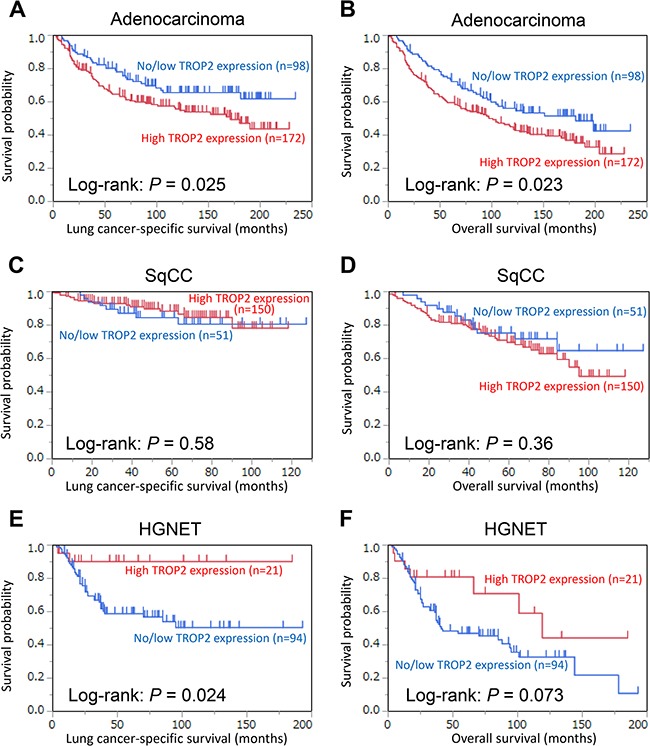
Kaplan–Meier curves for lung cancer-specific **(A, C, and E)** and overall survival **(B, D, and F)** according to TROP2 expression levels in tumor cells (no/low *vs*. high). (A and B) adenocarcinoma, (C and D) squamous cell carcinoma (SqCC), and (E and F) high-grade neuroendocrine tumor (HGNET).

Out of the 270 patients with adenocarcinoma, there were 149 deaths, including 109 lung cancer-specific deaths, during a median follow-up period of 13.0 years (IQR: 9.1–15.5 years) for the censored cases. High TROP2 expression was associated with shorter lung cancer-specific (log-rank, *P* = 0.025; Figure 2A) and overall survival (log-rank, *P* = 0.023; Figure 2B). A univariable Cox regression analysis revealed that high TROP2 expression was associated with shorter lung cancer-specific (univariable hazard ratio [HR] = 1.60, 95% confidence interval [CI] = 1.07–2.44, *P* = 0.022) and overall survival (univariable HR = 1.49, 95% CI = 1.06–2.13, *P* = 0.021). In a multivariable analysis, however, the association was not significant for both lung cancer-specific (*P* = 0.26) and overall survival (*P* = 0.21) (Table 2). Next, we analyzed the association of other covariates with patient mortality using univariable and multivariable Cox regression analyses. The results showed that p-stage (*P* < 0.0001), tumor differentiation grade (*P* = 0.0013), and age (*P* = 0.043) were confounding factors for TROP2 expression in lung cancer-specific survival (Supplementary Table 1).

**Table 2:** TROP2 expression and patient mortality^a^ in lung cancer

	Lung cancer-specific mortality						Overall mortality				
			Univariable analysis		Multivariable analysis^b^			Univariable analysis		Multivariable analysis^b^	
	*N* of cases	*N* of events	HR (95% CI)	*P*-values	HR (95% CI)	*P*-values	*N* of events	HR (95% CI)	*P*-values	HR (95% CI)	*P*-values
Adenocarcinoma				0.022		0.26			0.021		0.21
No/low TROP2 expression	98	32	1 (referent)		1 (referent)		46	1 (referent)		1 (referent)	
High TROP2 expression	172	77	1.60 (1.07-2.44)		1.27 (0.84-1.96)		103	1.49 (1.06 −2.13)		1.25 (0.88-1.80)	
SqCC				0.59		0.56			0.35		0.35
No/low TROP2 expression	51	8	1 (referent)		1 (referent)		13	1 (referent)		1 (referent)	
High TROP2 expression	150	17	0.79 (0.35-1.94)		0.78 (0.35-1.91)		46	1.34 (0.74-2.58)		1.33 (0.74-2.57)	
HGNET				0.0096		0.0003			0.057		0.0015
No/low TROP2 expression	94	38	1 (referent)		1 (referent)		55	1 (referent)		1 (referent)	
High TROP2 expression	21	2	0.23 (0.037-0.74)		0.13 (0.020-0.44)		7	0.50 (0.21-1.02)		0.30 (0.12-0.65)	

^a^Cox proportional hazards regression models were used to calculate HR and 95% CI.

^b^For adenocarcinoma, the multivariable model initially included age (< 60 years vs. ≥ 60 years), gender (male vs. female), smoking status (ever smoker vs. never smoker), tumor differentiation grade (well vs. moderate-poor), pathological stage (p-stage) (I vs. II–IV), *EGFR* status (wild type vs. mutant), *KRAS* status (wild type vs. mutant), and *ALK* rearrangement status (negative vs. positive). For SqCC, the multivariable model initially included age (< 60 years vs. ≥ 60 years), gender (male vs. female), smoking history (smoking index ≥ 400 vs. < 400), tumor differentiation grade (well vs. moderate-poor), and p-stage (I vs. II–IV). For HGNET, the multivariable model initially included age (< 60 years vs. ≥ 60 years), gender (male vs. female), smoking history (smoking index ≥ 400 vs. < 400), p-stage (I vs. II–IV), histology (SCLC vs. LCNEC), neoadjuvant chemotherapy (yes vs. no), and adjuvant chemotherapy (yes vs. no).

We created missing categories for any missing variables. A backward stepwise elimination with a threshold of *P* = 0.05 was performed to determine the variables for the final model.

Abbreviations: CI, confidence interval; HGNET, high-grade neuroendocrine tumor; HR, hazard ratio; p-stage, pathological stage; LCNEC, large cell neuroendocrine carcinoma; SCLC, small cell lung carcinoma; SqCC, squamous cell carcinoma.

Out of the 201 patients with SqCC, there were 59 deaths, including 25 lung cancer-specific deaths, during a median follow-up period of 5.0 years (3.1–6.3 years) for the censored cases. High TROP2 expression was not associated with lung cancer-specific (log-rank, *P* = 0.58; Figure 2C) or overall survival (log-rank, *P* = 0.36; Figure 2D). A Cox regression analysis revealed that high TROP2 expression was not associated with lung cancer-specific (univariable analysis, *P* = 0.59 and multivariable analysis, *P* = 0.56) or overall survival (univariable analysis, *P* = 0.35 and multivariable analysis, *P* = 0.35) (Table 2).

Out of the 115 patients with HGNET, there were 62 deaths, including 40 lung cancer-specific deaths, during a median follow-up period of 5.8 years (3.1–8.2 years) for the censored cases. High TROP2 expression was associated with longer lung cancer-specific (log-rank, *P* = 0.024; Figure 2E) and overall survival (log-rank, *P* = 0.073; Figure 2F). A univariable Cox regression analysis revealed that high TROP2 expression was associated with longer lung cancer-specific (univariable HR = 0.23, 95% CI = 0.037–0.74, *P* = 0.0096) and overall survival (univariable HR = 0.50, 95% CI = 0.21–1.02, *P* = 0.057). A multivariable analysis also showed that high TROP2 expression was associated with longer lung cancer-specific (multivariable HR = 0.13, 95% CI = 0.020–0.44, *P* = 0.0003) and overall survival (multivariable HR = 0.30, 95% CI = 0.12–0.65, *P* = 0.0015) (Table 2).

The association of other covariates with patient mortality for adenocarcinoma, SqCC, and HGNET using univariable and multivariable Cox regression analyses is also provided in Supplementary Table 1.

## DISCUSSION

We examined the association of TROP2 expression in tumors with clinicopathological/molecular features and with prognosis of various lung cancer subtypes, including adenocarcinoma, SqCC, and HGNET (SCLC and LCNEC). The association of high TROP2 expression with prognosis varied based on the lung cancer subtype. In adenocarcinoma, high TROP2 expression was associated with higher patient mortality. In SqCC, high TROP2 expression was not associated with mortality. In HGNET, high TROP2 expression was unexpectedly associated with lower patient mortality. This study suggests a differential role for TROP2 in different lung cancer subtypes.

Little is known about the association of TROP2 expression with clinicopathological/molecular features and prognosis in lung cancer subtypes. Kobayashi et al. reported that TROP2 overexpression was associated with higher overall mortality in 130 patients with small-sized (< 2cm) lung adenocarcinoma (*P* = 0.056) [1]. Li et al. showed an association of high TROP2 expression with poor prognosis (*P* = 0.046) in 68 cases of adenocarcinoma and demonstrated that TROP2 overexpression enhanced cell proliferation, migration, and invasion in the lung adenocarcinoma cell line A549 [3]. In contrast, Pak et al. reported that TROP2 overexpression resulted in a better overall survival in 100 patients with lung adenocarcinoma (*P* = 0.02) and showed a tendency toward better overall survival in 64 patients with SqCC (*P* = 0.49) [2]. To the best of our knowledge, our study is the first to examine the prognostic association of TROP2 expression in HGNET (SCLC and LCNEC). In addition, our study used the largest sample sizes of lung adenocarcinoma and SqCC to examine TROP2 expression. We demonstrated that high TROP2 expression was related to differential prognoses based on the lung cancer subtype. High TROP2 expression was associated with higher mortality in lung adenocarcinoma, was not associated with mortality in SqCC, and was associated with lower mortality in HGNET. Similarly, PD-L1 positivity was associated with higher mortality in lung adenocarcinoma [29–31], whereas PD-L1 positivity was related to lower mortality in SCLC [32, 33]. Thus, PD-L1 and TROP2 appear to play different roles depending on the lung cancer subtype. Of interest, high TROP2 expression was significantly more frequent in LCNEC than in SCLC. Emerging evidence suggests that LCNEC is a biologically heterogeneous group, containing SCLC-phenotype/NSCLC-phenotype [34] and YAP1-negative group/YAP1-positive group[35]. There may be an association of high TROP2 expression in LCNEC with SCLC-phenotype/NSCLC-phenotype or YAP1 expression. Because the Ki-67 index was not substantially different according to the TROP2 expression level (no/low vs. high) in HGNET, the other mechanisms except for proliferation, including SCLC-phenotype/NSCLC-phenotype or YAP1 expression, might explain the differential clinicopathological differences according to the TROP2 expression level in HGNET. The use of different downstream signaling pathways may explain the divergent associations of TROP2 expression with prognosis for adenocarcinoma and SqCC [36]. Further studies are required to elucidate the mechanisms accounting for differential clinicopathological associations according to tumor histological subtypes.

We also determined the prevalence of high TROP2 expression and its association with clinicopathological/molecular features in different lung cancer subtypes. A high proportion of patients with adenocarcinoma (64%) and SqCC (75%) showed high TROP2 expression, suggesting that therapies targeting TROP2 may be effective. However, a low proportion of high TROP2 expression was observed in HGNET tumors (18%), suggesting that the probability of good clinical response of HGNET to this type of therapy may be low, although that of TROP2-expressing HGNET may be high. In adenocarcinoma, high TROP2 expression was associated with the male gender, larger tumor size, advanced stage, and less tumor differentiation but not with genetic alterations in *EGFR*, *KRAS*, or *ALK*. In HGNET, 34% (14/41) of LCNEC and 9.5% (7/74) of SCLC tumors showed high TROP2 expression. This information must be useful for the development of therapies targeting TROP2.

A growing body of evidence suggests that TROP2 is a promising molecular target for the treatment of various malignancies [11]. IMMU-132 is an anti-TROP2 ADC that has been shown to be effective against various cancers without severe side effects in various cancers [11–18], including metastatic SCLC [14] and NSCLC resistant to anti-PD-1/PD-L1 therapy [15]. Because little data are available on TROP2 expression in lung cancer, our data are valuable for establishing the utility of TROP2-targeting therapies.

Our study had limitations that need to be stated. First, there is no standardized method for the immunohistochemical assessment of TROP2 expression in tumors, which may influence the reproducibility of the results. We evaluated membranous TROP2 expression in cancer cells because this assessment is required to predict the efficacy of molecular-targeted therapies for lung cancer. However, some studies evaluated both membranous and cytoplasmic TROP2 expression, which could yield conflicting results as to the association of TROP2 expression with clinicopathological/prognostic features. In our study, two pathologists conducted a blinded and independent assessment of TROP2 expression with a good interobserver agreement. Second, we used tissue microarrays to evaluate TROP2 expression in tumors. Intratumoral heterogeneity is a characteristic of lung cancer; thus, tumors with heterogeneous TROP2 expression can affect the results. We speculate that this potential misclassification of tumors based on TROP2 expression would be randomly dispersed; therefore, null results would have been yielded. Nonetheless, we have shown statistically significant results. In addition, an experienced pulmonary pathologist (KI) chose each core site with a relatively large diameter (2 mm) based on the most histologically representative region of the tumor to minimize the chance of this potential misclassification affecting the results. Third, the difference of follow-up periods among the three subtypes might be a potential confounder of our results. Nonetheless, we monitored survivors for the median (interquartile range) of 13.0 years (9.1–15.5 years) in adenocarcinoma, 5.0 years (3.1–6.3 years) in SqCC, and 5.8 years (3.1–8.2 years) in HGNET. Therefore, less time between surgery and death in SqCC and HGNET did not appear to affect our results substantially. Fourth, the total number of patients, especially those with HGNET (*N* = 115), was not sufficient, and the statistical power was therefore limited. Fifth, our database was retrospectively created. Finally, we only enrolled Japanese patients at a single cancer hospital. Therefore, additional studies in other patient populations are needed.

In conclusion, we demonstrated that the prognostic association of high TROP2 expression differed according to lung cancer subtypes. Although high TROP2 expression was associated with higher mortality in lung adenocarcinoma, it was associated with lower mortality in HGNET, and was not associated with mortality in SqCC. We also determined the prevalence of high TROP2 expression and its association with clinicopathological/molecular features in these lung cancer subtypes. This information is beneficial for determining the utility of TROP2-targeting therapy. Additional large-scale studies are required to confirm our findings.

## MATERIALS AND METHODS

### Study population

We examined 270 consecutive cases of lung adenocarcinoma, 201 cases of SqCC, and 115 cases of HGNET (74 SCLC cases and 41 LCNEC cases) to assess the TROP2 expression in tumors and survival. Lung adenocarcinoma, SqCC, and HGNET were surgically resected between April 1995 and January 2002, between April 2005 and February 2014, and between July 1990 and November 2014, respectively, at The Cancer Institute Hospital, Japanese Foundation for Cancer Research (JFCR) in Tokyo, Japan. Patients were observed until death or December 1, 2015. For the assessment of smoking history, we used a smoking index (SI) calculated by multiplying the “number of cigarettes per day” by “duration in years.” This study was approved by the institutional review board of JFCR, and informed consent was obtained from all patients included in this study.

### Pathological evaluation

Pathological diagnoses were made by experienced expert pulmonary pathologists (KI and YI), essentially based on the 2015 WHO classification of lung tumors [37]. Tumor differentiation grades were defined according to the Japanese Lung Cancer Society criteria [38, 39]. All patients were pathologically staged according to the 7^th^ edition of the AJCC-TNM staging system [40].

### Immunohistochemistry for TROP2 and Ki-67

Membranous TROP2 expression of tumor cells was evaluated by an immunohistochemical analysis of tissue microarrays. Using the archived surgical specimens used for initial pathological diagnoses of primary lung cancers, we constructed tissue microarrays as previously described [41]. Briefly, we punched points of the donor paraffin blocks using a 2 mm-diameter coring needle and transferred the tissue to the array in the recipient block using a manual tissue arrayer (KIN-1; Azumaya, Tokyo, Japan). For each tumor, an experienced pulmonary pathologist (KI) selected one site exhibiting the most representative histology for that tumor [42].

Sections with a thickness of 4 μm were immunostained for TROP2 with an anti-TROP2 mouse monoclonal antibody (clone: 1E5-1E2, Daiichi Sankyo Co., Ltd., Tokyo, Japan; diluted 1:400) using the Leica Bond III automated system (Leica Biosystems Melbourne Pty Ltd., Australia). This monoclonal antibody recognizes an epitope in the extracellular domain (Mer1-Thr274) of human TROP2. The sections were incubated at pH 6 for 10 min at 100°C. TROP2 expression on the membranes of tumor cells was interpreted by an experienced pulmonary pathologist (KI) in a blinded manner. The intensity of TROP2 membranous staining in tumor cells was defined as 0 (absent), 1 (weak to moderate), or 2 (strong) (Figure 1). We calculated the percentage of tumor cells at each TROP2 intensity level. For the statistical analyses, we categorized the specimens into two groups based on the staining intensity and percentage of positive cells: no/low TROP2 expression (Intensity 1 < 50% and Intensity 2 < 10%) and high TROP2 expression (Intensity 1 ≥ 50% or Intensity 2 ≥ 10%). A random sample set of 127 cases of lung adenocarcinoma, all 201 cases of SqCC, and all 115 cases of HGNET were blindly examined by a second pathologist (YY). There were high concordances between the two observers, as evidenced by a kappa of 0.66 (95% CI = 0.53–0.79; *P* < 0.0001) for adenocarcinoma, 0.60 (95% CI = 0.47–0.73;*P* < 0.0001) for SqCC, and 0.63 (95% CI = 0.45–0.81; *P* < 0.0001) for HGNET. In addition, to assess intratumoral heterogeneity of TROP2 expression, we immunostained TROP2 using whole sections from 20, 15, and 12 cases of adenocarcinoma, SqCC, and HGNET (6 SCLCs and 6 LCNECs), respectively and did not observe substantial intratumoral heterogeneity in any cases in terms of tumor TROP2 expression.

For HGNET, we also conducted immunostaining for Ki-67 (MIB-1, Dako, Glostrup, Denmark; diluted 1:200) using the Leica Bond III automated system (Leica Biosystems Melbourne Pty Ltd.). The tumor Ki-67 index was calculated by an experienced pulmonary pathologist (KI) in a blinded manner. For the statistical analysis, we categorized the specimens into two groups by the Ki-67 index of 60%, which is a median of all HGNET cases (< 60% vs. ≥ 60%). All 115 cases of HGNET were blindly examined by a second pathologist (YY) with a high concordance between the two observers, as evidenced by a kappa of 0.66 (95% CI = 0.53–0.79; *P* < 0.0001).

For negative and positive controls, we used a cell array (provided by Daiichi Sankyo Co., Ltd., Tokyo, Japan). This cell array consisted of cell line Calu-6 (ATCC, Manassas, VA, USA) showing very low (below the detection limit) TROP2 expression and N-87 (ATCC) showing high TROP2 expression. Immunohistochemistry was performed using Calu-6 as a specific negative control and N-87 as a positive control. Sections processed with replacement of primary antibody by Dako REAL™ Antibody Diluent (Dako, Glostrup, Denmark) were also used as a non-specific negative control.

### Detection of *EGFR* and *KRAS* mutations and *ALK* fusion

Tumor specimens were snap-frozen in liquid nitrogen typically within 20 min after surgical resection and stored at −80°C until use. DNA was extracted using a standard proteinase K digestion and phenol-chloroform extraction. For analysis of the *EGFR* mutation, we examined four exons (exons 18–21) that code for the tyrosine kinase domain of the *EGFR* gene. For exons 18 (G719X), 20 (S768I and T790M), and 21 (L858R and L861Q), the TaqMan™ SNP Genotyping Assay kit (Applied Biosystems, Foster City, CA, USA) was used according to the manufacturer's instructions. For exon 19 deletion and exon 20 insertion, a fragment analysis was conducted, as previously described [43]. For the analysis of *KRAS* mutation, we performed direct sequencing assays for codons 12, 13, and 61, as previously described [43].

For detection of *ALK* fusion, we performed immunohistochemistry using an anti-ALK mouse monoclonal antibody (clone: 5A4, Leica Biosystems Newcastle Ltd., UK; diluted 1:50) and the Leica Bond III automated system (Leica Biosystems Melbourne Pty Ltd). The sections were incubated at pH 9 for 30 min at 100°C. In the ALK-positive tumors, *ALK* fusions were confirmed by fluorescence *in situ* hybridization, as previously described [27].

### Statistical analysis

All statistical analyses were conducted using the JMP statistical software package 12 (SAS Institute Inc., Cary, NC, USA) and Excel 2013 software (Microsoft, Redmond, WA, USA). All *P*-values were two-sided. The statistical significance level was set to *P* = 0.05.

To investigate the association of TROP2 expression with clinicopathological and molecular features in lung cancer, we used the chi-square or Fisher's exact test as appropriate.

The Kaplan–Meier method and log-rank test were used for survival analyses. For the analysis of lung cancer-specific mortality, deaths as a result of other causes were censored. We also used univariable and multivariable Cox proportional hazards regression models to calculate HR for mortality according to the TROP2 expression level. For adenocarcinoma, the multivariable model initially included age (< 60 years vs. ≥ 60 years), gender (male vs. female), smoking status (ever smoker vs. never smoker), tumor differentiation grade (well vs. moderate-poor), p-stage (I vs. II–IV), *EGFR* status (wild type vs. mutant), *KRAS* status (wild type vs. mutant), and *ALK* rearrangement status (negative vs. positive). For SqCC, the multivariable model initially included age (< 60 years vs. ≥ 60 years), gender (male vs. female), smoking history (smoking index ≥ 400 vs. < 400), tumor differentiation grade (well vs. moderate-poor), and p-stage (I vs. II–IV). For HGNET, the multivariable model initially included age (< 60 years vs. ≥ 60 years), gender (male vs. female), smoking history (smoking index ≥ 400 vs. < 400), p-stage (I vs. II–IV), histology (SCLC vs. LCNEC), neoadjuvant chemotherapy (yes vs. no), and adjuvant chemotherapy (yes vs. no). We created missing categories for any missing variable, if applicable. A backward stepwise elimination was performed using a *P* = 0.05 threshold to select variables for the final model. The proportionality of hazards assumption in each subtype was confirmed using the graphs of the log(−log[survival probability]) vs. log of survival time to visually assess if the lines were approximately parallel.

## SUPPLEMENTARY MATERIALS TABLE





## Abbreviations

ADC: antibody-drug conjugate
CI: confidence interval
HGNET: high-grade neuroendocrine tumor
HR: hazard ratio
IQR: interquartile range
LCNEC: large cell neuroendocrine carcinoma
NSCLC: non-small cell lung carcinoma
p-stage: pathological stage
SCLC: small cell lung carcinoma
SI: smoking index
SqCC: squamous cell carcinoma.
