# Supplementary material for: Association of tumor TROP2 expression with prognosis varies among lung cancer subtypes
Source: Oncotarget. 2017 Feb 23;8(17):28725–35. doi: 10.18632/oncotarget.15647 (PMC5438686; doi:10.18632/oncotarget.15647)
Supplement: Supplementary file 1 [file oncotarget-08-28725-s001.pdf]

Association of tumor TROP2 expression with prognosis varies among lung cancer subtypes

SUPPLEMENTARY TABLE

Supplementary Table 1: Covariates and patient mortality[a] in lung cancer.

See Supplementary File 1
