# Supplementary material for: Association of tumor TROP2 expression with prognosis varies among lung cancer subtypes
Source: Oncotarget. 2017 Feb 23;8(17):28725–35. doi: 10.18632/oncotarget.15647 (PMC5438686; doi:10.18632/oncotarget.15647)
Supplement: Supplementary file 2 [file oncotarget-08-28725-s002.docx]

**Supplementary Table 1. Covariates and patient mortality^a^ in lung cancer**

|  | | Lung cancer-specific mortality | | | |  | Overall mortality | | | |
| --- | --- | --- | --- | --- | --- | --- | --- | --- | --- | --- |
|  |  | Univariable analysis | | Multivariable analysis^b^ | |  | Univariable analysis | | Multivariable analysis^b^ | |
|  |  | HR  (95% CI) | *P*-values | HR  (95% CI) | *P*-values |  | HR  (95% CI) | *P*-values | HR  (95% CI) | *P*-values |
| **Adenocarcinoma** | |  |  |  |  |  |  |  |  |  |
|  | TROP2 expression: high  (vs. no/low) | 1.60  (1.07-2.44) | 0.022 | 1.27  (0.84-1.96) | 0.26 |  | 1.49  (1.06 -2.13) | 0.021 | 1.25  (0.88-1.80) | 0.21 |
|  | p-stage: II–IV  (vs. I) | 5.52  (3.66-8.55) | <0.0001 | 4.34  (2.81-6.87) | <0.0001 |  | 3.67  (2.63-5.16) | <0.0001 | 2.91  (2.04-4.18) | <0.0001 |
|  | Differentiation: moderate to poor  (vs. well) | 3.51  (2.28-5.61) | <0.0001 | 2.11  (1.33-3.46) | 0.0013 |  | 3.23  (2.24-4.75) | <0.0001 | 2.24  (1.52-3.37) | <0.0001 |
|  | Age (years): ≥ 60  (vs. < 60) | 1.27  (0.86-1.91) | 0.24 | 1.50  (1.01-2.26) | 0.043 |  | 1.61  (1.14-2.31) | 0.0068 | 1.83  (1.29-2.63) | 0.0006 |
|  | *EGFR* status: wild type  (vs. mutant) | 1.49  (0.96-2.31) | 0.074 |  |  |  | 1.53  (1.06-2.24) | 0.025 |  |  |
|  | Gender: male  (vs. female) | 1.38  (0.94-2.03) | 0.099 |  |  |  | 1.57  (1.14-2.20) | 0.0064 |  |  |
|  | Smoking habit: ever smoker  (vs. never) | 1.16  (0.79-1.71) | 0.45 |  |  |  | 1.39  (1.00-1.96) | 0.049 |  |  |
|  | *KRAS* status: mutant type  (vs. wild) | 1.13  (0.55-2.08) | 0.72 |  |  |  | 1.37  (0.78-2.26) | 0.26 |  |  |
|  | *ALK* rearrangement: negative  (vs. positive) | 1.09  (0.46-3.57) | 0.86 |  |  |  | 1.54  (0.65-5.01) | 0.36 |  |  |
|  | |  |  |  |  |  |  |  |  |  |
| **SqCC** | |  |  |  |  |  |  |  |  |  |
|  | TROP2 expression: high  (vs. no/low) | 0.79  (0.35-1.94) | 0.59 | 0.78  (0.35-1.91) | 0.56 |  | 1.34  (0.74-2.58) | 0.35 | 1.33  (0.74-2.57) | 0.35 |
|  | p-stage: II–IV  (vs. I) | 6.23  (2.52-18.7) | <0.0001 | 6.24  (2.53-18.8) | <0.0001 |  | 2.46  (1.47-4.20) | 0.0006 | 2.46  (1.46-4.20) | 0.0006 |
|  | Smoking index: < 400  (vs. ≥ 400) | 1.95  (0.46-5.68) | 0.32 |  |  |  | 1.11  (0.33-2.70) | 0.85 |  |  |
|  | Differentiation: moderate to poor  (vs. well) | 2.22  (0.47-39.7) | 0.38 |  |  |  | 1.01  (0.44-2.89) | 0.99 |  |  |
|  | Gender: female  (vs. male) | 1.75  (0.52-10.9) | 0.41 |  |  |  | 1.62  (0.72-4.66) | 0.27 |  |  |
|  | Age (years): ≥ 60  (vs. < 60) | 1.01  (0.34-4.28) | 0.99 |  |  |  | 1.94  (0.79-6.42) | 0.16 |  |  |
|  | |  |  |  |  |  |  |  |  |  |
| **HGNET** | |  |  |  |  |  |  |  |  |  |
|  | TROP2 expression: high  (vs. no/low) | 0.23  (0.037-0.74) | 0.0096 | 0.13  (0.020-0.44) | 0.0003 |  | 0.50  (0.21-1.02) | 0.057 | 0.30  (0.12-0.65) | 0.0015 |
|  | p-stage: II–IV  (vs. I) | 2.67  (1.37-5.59) | 0.0034 | 4.12  (2.03-8.91) | <0.0001 |  | 1.61  (0.97-2.75) | 0.067 | 2.31  (1.34-4.06) | 0.0024 |
|  | Adjuvant chemotherapy: no  (vs. yes) | 1.88  (1.01-3.53) | 0.048 | 3.77  (1.90-7.49) | 0.0002 |  | 2.13  (1.29-3.54) | 0.0033 | 3.61  (2.05-6.41) | <0.0001 |
|  | Neoadjuvant chemotherapy: yes  (vs. no) | 2.08  (0.99-4.05) | 0.053 | 2.36  (1.10-4.72) | 0.028 |  | 2.11  (1.15-3.67) | 0.017 | 2.32  (1.25-4.09) | 0.0085 |
|  | Smoking index: < 400  (vs. ≥ 400) | 1.59  (0.71-3.21) | 0.24 |  |  |  | 1.30  (0.66-2.37) | 0.43 |  |  |
|  | Histology: SCLC  (vs. LCNEC) | 1.21  (0.64-2.42) | 0.57 |  |  |  | 1.13  (0.68-1.95) | 0.64 |  |  |
|  | Age (years): ≥ 60  (vs. < 60) | 1.20  (0.61-2.59) | 0.61 |  |  |  | 1.73  (0.95-3.40) | 0.074 |  |  |
|  | Gender: female  (vs. male) | 1.12  (0.53-2.76) | 0.78 |  |  |  | 1.05  (0.53-1.91) | 0.88 |  |  |

^a^Cox proportional hazards regression models were used to calculate HR and 95% CI.

^b^For adenocarcinoma, the multivariable model initially included age (< 60 years vs. ≥ 60 years), gender (male vs. female), smoking status (ever smoker vs. never smoker), tumor differentiation grade (well vs. moderate-poor), pathological stage (p-stage) (I vs. II–IV), *EGFR* status (wild type vs. mutant), *KRAS* status (wild type vs. mutant), and *ALK* rearrangement status (negative vs. positive). For SqCC, the multivariable model initially included age (< 60 years vs. ≥ 60 years), gender (male vs. female), smoking history (smoking index ≥ 400 vs. < 400), tumor differentiation grade (well vs. moderate-poor), and p-stage (I vs. II–IV). For HGNET, the multivariable model initially included age (< 60 years vs. ≥ 60 years), gender (male vs. female), smoking history (smoking index ≥ 400 vs. < 400), p-stage (I vs. II–IV), histology (SCLC vs. LCNEC), neoadjuvant chemotherapy (yes vs. no), and adjuvant chemotherapy (yes vs. no).

We created missing categories for any missing variables. A backward stepwise elimination with a threshold of *P* = 0.05 was performed to determine the variables for the final model.

CI, confidence interval; HGNET, high-grade neuroendocrine tumor; HR, hazard ratio; p-stage, pathological stage; LCNEC, large cell neuroendocrine carcinoma; SCLC, small cell lung carcinoma; SqCC, squamous cell carcinoma.
